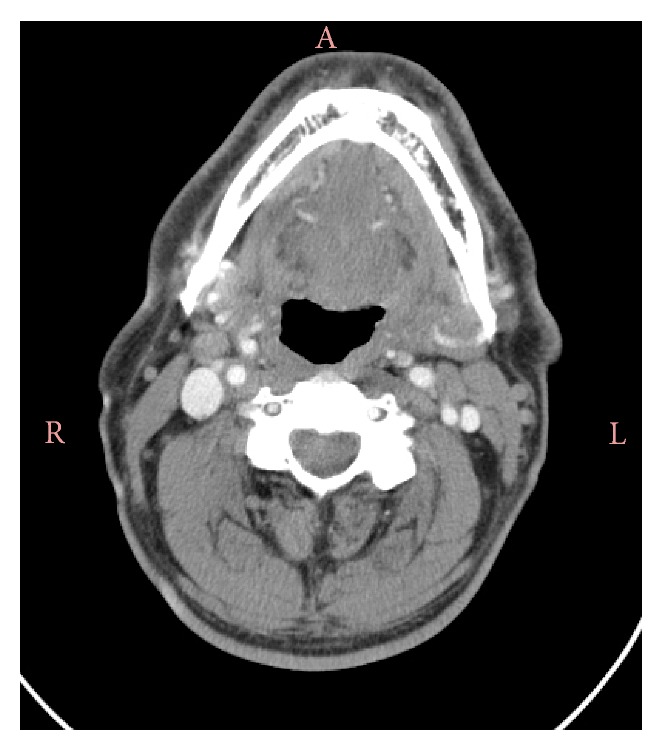# A Rare Cause of Acute Dysphagia: Abscess of the Base of the Tongue

**DOI:** 10.1155/2015/431738

**Published:** 2015-02-23

**Authors:** Gulsum Teke Ozgur, Mehmet Volkan Akdogan, Gulhan Kanat Unler, Huseyin Savas Gokturk

**Affiliations:** ^1^Department of Family Medicine, Baskent University Faculty of Medicine, Konya, Turkey; ^2^Baskent University Faculty of Medicine, Konya, Turkey; ^3^Department of Gastroenterology, Baskent University, Hoca Cihan Mah Saray Caddesi, No. 1, Selcuklu, 42080 Konya, Turkey

## Abstract

Dysphagia represents a difficulty in passage of solid or liquid foods from the oral cavity into the stomach and is considered as an alarm symptom of gastrointestinal system. It often indicates an organic disease and needs to be explained. In this paper, a case of 61-year-old man with posterior tongue abscess is presented.

## 1. Introduction

Dysphagia represents a difficulty in passage of solid or liquid foods from the oral cavity into the stomach and is considered as an alarm symptom of gastrointestinal system. It often indicates an organic disease and needs to be explained. Dysphagia is classified into two major types: oropharyngeal (high) dysphagia and esophageal dysphagia based on the localization [1, 2].

Oropharyngeal dysphagia is characterized by difficulty in initiating swallowing and usually indicates a problem on the oral or pharyngeal localization. Although the most frequent causes of oropharyngeal dysphagia are central nervous system disorders in older people and inflammatory muscle diseases in young, the infections of oral cavity and the neck should be remembered. Although tongue abscesses are rarely seen, they should be kept in mind in differential diagnosis of dysphagia. If the early diagnosis and treatment of posterior tongue abscesses is not achieved, it may lead to sudden life-threatening respiratory complications.

In this paper, a case of 61-year-old man with posterior tongue abscess is presented with clinical, radiological, and microbiological features. Taking a careful history, the appropriate diagnostic tests, and consultations with other departments and planning an immediate surgical and/or medical treatment will follow.

## 2. Case Report

**Figure 1:**
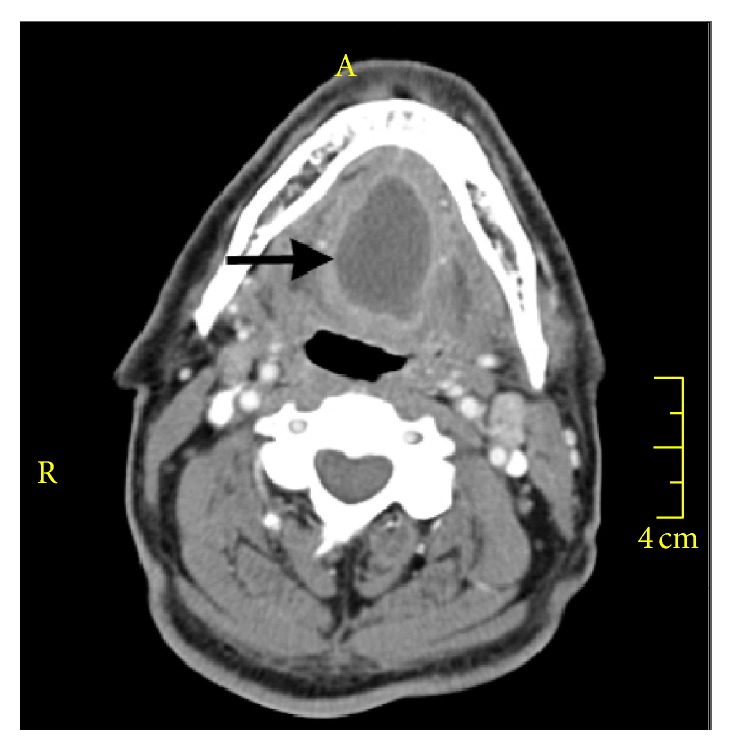


A 61-year-old man was admitted to the department of the gastroenterology with complaints of odynophagia and dysphagia to solid foods for one month. He experienced worsening of dysphagia and he also reported otalgia for the last two days. His oral intake had decreased and he had preferred to consume liquids. As a result, he had lost 10 kg in a month. Fifteen days before the admission, the patient was assessed by several ENT outpatient clinics and was treated for pharyngitis with two different antibiotics. He had no history of smoking, alcohol consumption, or any other systemic diseases. During the questioning of the patient, he pointed out his neck region as the site of obstruction. On physical examination, he was afebrile with normal vital signs. However, the palpation of the neck was painful. The white blood cell count was 6,940/mm³, C-reactive protein was 51 mg/L, and erythrocyte sedimentation rate was 64 mm/hour. The standard barium swallow study was normal. The upper gastrointestinal endoscopy revealed normal findings except for erosive bulbitis. The etiology of dysphagia had not been identified by these tests and a computed tomography (CT) scan of the neck was performed. The CT scan demonstrated a 4 × 2.5 cm sized cystic lesion with minimal irregular border at the base of the tongue (Figure 1). The patient was referred to the ENT department. On his ENT examination, there was a slight swelling at the base of the tongue. The larynx and the tonsils were normal. There were no signs of airway obstruction. The abscess at the base of the tongue was drained through the oral route by needle aspiration for five consecutive days. On the first day, a 15 cc purulent material was drained and the aspirated material was cultured. Coagulase-negative staphylococci were isolated from the culture media and the empirical treatment with ceftriaxone based on the antibiogram results was not changed. The patient was ordered to complete a 7- to 10-day course of oral antibiotic therapy. The follow-up CT scan of neck performed one month later revealed complete disappearance of the abscess (Figure 2).

## 3. Discussion

Oropharyngeal dysphagia, cervical dysphagia, and transfer dysphagia are synonyms of each other. The cricopharyngeal region and the upper 1/4 of esophagus are under the direct control of the central nervous system. The pharyngeal phase of swallowing is under the management of brain stem and controlled reflexively.

The causes of oropharyngeal dysphagia can be classified as either the neuromuscular or the structural. The most commonly seen neuromuscular pathologies are cerebrovascular accidents, Parkinson's disease, Myasthenia gravis (affecting nerve-muscle junction), and polymyositis (affecting muscles). Carcinomas, cervical osteophytes, esophageal webs, radiation and surgical therapies, Zenker's diverticulum, Schatzki's rings, peptic strictures, goiter, infections of the neck, and pharynx are the structural causes [2, 3].

Although the tongue is exposed to many potential pathogens, it is resistant to infections. Thus, tongue abscesses are rarely seen infections due to the strong protective mechanism. The protective mechanisms are the increased vascularization and lymphatic drainage, thick keratinized mucosa of the tongue, the immunological properties of saliva, and the constant mobility of the tongue which reveals cleaning effect of saliva [4]. The anterior two-thirds of the tongue is referred to as the oral tongue. This part is the freely moving part and lies anterior to the circumvallate papillae. The part lying posterior to the circumvallate papillae is referred to as tongue base and is regarded as a part of the oropharynx. The tongue abscesses are classified in two groups: anterior tongue abscess and posterior third tongue abscess. The etiologies of the tongue abscess vary according to its localization. Posterior abscesses are rarely seen and are usually derived from lingual tonsillitis, infected thyroglossal duct cyst remnants, and periodontal infections spreading from lower molar teeth [4, 5]. The predisposing factors of the tongue abscess are poor oral hygiene, immunodeficiency status, chemotherapeutic drugs, and the diabetes. Some case studies revealed that immunodeficient state is considered a predisposing risk factor for the development of tongue abscess [4, 6]. Sánchez Barrueco et al. presented a recurrent tongue abscess case with a history of diabetes and tongue laceration [7]. Evaluation of underlying medical problem and history of trauma to tongue were essential in the diagnosis of tongue abscesses. However, in some cases, no specific cause can be found, as in our case [8].

The symptoms of the tongue abscess are sudden painful swelling of the tongue within hours or days, pain radiating toward the ears, and voluntary fixation of tongue due to pain, fever, dyspnea, dysphagia, and odynophagia. Dysphagia and dyspnea are the alarm symptoms that the lesion should be decompressed urgently and airway maintenance is necessary. Therefore, ENT surgeons must be consulted in time to prevent life-threatening results. Patients with oropharyngeal dysphagia usually report discomfort in the cervical region [6, 9]. Our patient was also pointing to his neck while questioning. Dysphagia, odynophagia, and otalgia were the most prominent symptoms in our patient in the last days. Otalgia and dysphagia relieved immediately after the abscess drainage by ENT specialist.

The differential diagnosis for the swellings of the tongue includes carcinomas, anaphylaxis, acute epiglottitis, dermoid cysts, lipoma, lingual artery aneurysm, arteriovenous malformation, infarction, hemorrhage, lingual tonsillitis, thyroglossal cysts, tuberculosis, and actinomycosis [9]. The diagnosis of tongue abscess is not always easy, as in our case [9, 10]. The symptoms and signs vary widely. In cases where the clinical findings were insignificant, laboratory and radiological tests may be more helpful. Ultrasonography, computed tomography, and magnetic resonance imaging are generally recommended for differential diagnosis of tongue swellings [7, 10].

The successful treatment of tongue abscess begins with an accurate diagnosis and consists of airway maintenance, abscess drainage, and antibiotic treatment. It has been reported in the literature that the surgical drainage of the posterior tongue abscess may be technically difficult. Drainage may cause edema and respiratory involvement; thus the general anesthesia and endotracheal intubation may be required. Vellin et al. and Balatsouras et al. preferred needle aspiration technique in their case reports [5, 8]. To avoid airway compromise we also preferred this more conservative method instead of incision and drainage in the operating room. The patient was effectively treated by intraoral needle aspiration followed by antibiotic treatment without the need of hospitalization.

## 4. Conclusion

The posterior tongue abscesses are rare but potentially life-threatening pathologies. Trauma, foreign bodies, dental infections, tonsillitis, and surgical history are the most common etiological factors. If there is dysphagia to solids, which is unexplained by barium swallow pharyngoesophagography and upper endoscopic examinations, tongue abscesses should be considered and it should be noted that the neck CT may also help in the diagnosis.

## Figures and Tables

**Figure 2:**